# Geographical variation in the risk of H7N9 human infections in China: implications for risk-based surveillance

**DOI:** 10.1038/s41598-020-66359-1

**Published:** 2020-06-25

**Authors:** Xiaoyan Zhou, Lu Gao, Youming Wang, Yin Li, Yi Zhang, Chaojian Shen, Ailing Liu, Qi Yu, Wenyi Zhang, Alexander Pekin, Fusheng Guo, Carl Smith, Archie C. A. Clements, John Edwards, Baoxu Huang, Ricardo J. Soares Magalhães

**Affiliations:** 1 School of Veterinary Science, The University of Queensland, Brisbane, Australia; 2 China Animal Health and Epidemiology Centre, Ministry of Agriculture and Rural Affairs, Qingdao, PR China; 3 School of Veterinary and Biomedical Sciences, Murdoch University, Perth, Australia; 4 Beijing Center for Animal Disease Prevention and Control, Beijing, PR China; 5 Institute of Disease Control and Prevention, Academy of Military Medical Science, Beijing, PR China; 6 Food and Agriculture Organization of the United Nations (FAO), Bangkok, Thailand; 7 School of Business, The University of Queensland, Brisbane, Australia; 8 Faculty of Health Sciences, Curtin University, Perth, Australia; 9 Telethon Kids Institute, Perth, Australia; 10 Child Health Research Centre, The University of Queensland, Brisbane, Australia

**Keywords:** Ecological modelling, Influenza virus, Risk factors

## Abstract

The influenza A (H7N9) subtype remains a public health problem in China affecting individuals in contact with live poultry, particularly at live bird markets. Despite enhanced surveillance and biosecurity at LBMs H7N9 viruses are now more widespread in China. This study aims to quantify the temporal relationship between poultry surveillance results and the onset of human H7N9 infections during 2013–2017 and to estimate risk factors associated with geographical risk of H7N9 human infections in counties in Southeast China. Our results suggest that poultry surveillance data can potentially be used as early warning indicators for human H7N9 notifications. Furthermore, we found that human H7N9 incidence at county-level was significantly associated with the presence of wholesale LBMs, the density of retail LBMs, the presence of poultry virological positives, poultry movements from high-risk areas, as well as chicken population density and human population density. The results of this study can influence the current AI H7N9 control program by supporting the integration of poultry surveillance data with human H7N9 notifications as an early warning of the timing and areas at risk for human infection. The findings also highlight areas in China where monitoring of poultry movement and poultry infections could be prioritized.

## Introduction

Since the emergence in early 2013 of a low pathogenic avian influenza (LPAI) H7N9 virus^1^, there have been six epidemic waves causing about 1,600 human infections in 29 provinces and municipalities in mainland China^2^. During the fifth epidemic wave starting in October 2016, the geographic range of H7N9 human cases expanded and more human cases were reported than any previous wave^3^. In February 2017, strains of the 2013 LPAI H7N9 virus isolated from chickens in Guangdong province mutated to become highly pathogenic avian influenza (HPAI) H7N9 in poultry and rapidly spread to other provinces of China^4,5^. The rapid evolution, increased pathogenicity and transmissibility of HPAI H7N9 viruses in mammalian models, together with their extended host range, may have increased the threat to public health and the poultry industry^6,7^.

Live bird markets (LBMs) remain the main source of H7N9 virus spreading among poultry, and from poultry to humans^8^. Recognizing the role of LBMs in the exposure and dissemination of H7N9 viruses, in Feb 2017, the Ministry of Agriculture and Rural Affairs (MARA) of China established the “1110 policy”, which includes mandatory daily market cleaning activities, disinfection, market closure once a month, and no overnight market poultry storage. This policy was followed in July 2017, by the implementation of the National Vaccination Program in the poultry sector through the adoption of a bivalent H5/H7 inactivated vaccine. While this vaccine has largely been effective at controlling H7N9 virus circulation among both chicken and humans^5,7,9^, the virus is still being occasionally detected by the national animal disease surveillance system^10^. Therefore, a better understanding of the determinants of exposure is necessary to complement sanitary measures such as vaccination and enhanced LBM biosecurity.

The available literature indicates that the primary risk factor for human H7N9 infection in China is exposure to LBMs, and that intervention at this stage of the live poultry market chain is the most effective prevention measure^11–17^. Poultry-to-human transmission is intensified at LBMs, hence as a short term response, LBM closure should be rapidly implemented in areas where the virus is identified in either poultry or humans^18,19^. However, this may not be favorable to poultry enterprises or individual households due to the associated financial costs. Reactive closure of LBMs may facilitate further dissemination through the opening of unregistered LBMs or illegal poultry movements^20^.

Surveillance and monitoring of avian influenza within the poultry market chain (i.e. farms, live bird markets and slaughter houses) generates epidemiological evidence on affected species, geographical sources of infection and the role of modifiable risk factors on disease transmission^21^. Animal health authorities in China have been prompt at identifying the presence of the H7N9 virus within the live poultry market chain and controlling infection transmission at the source since the emergency. The control of H7N9 in chickens through vaccination explains the sudden decrease in the number of human H7N9 infections since October 2017^7,9^. Little is known about the relative timing of infections in people and poultry, which should peaks in transmission in poultry and precede human cases. Poultry surveillance results could provide an early warning for the likely timing and location of human H7N9 infections, however, this requires further evaluation. Furthermore, the role of poultry movements from the originally affected area in Eastern China in disseminating H7N9 infection throughout the country is yet to be quantified.

Several ecological spatial studies aiming at identifying risk factors of H7N9 human cases have been undertaken in China^3,22–26^, and distribution of H7N9 risks were mapped in these studies. Of these, two studies by Fuller *et al*. and Gilbert *et al*. attempted to map the suitability for H7N9 human infections in Asian regions. LBM density was demonstrated to be significantly associated with the presence of human H7N9 infections^3,23,26^. Human population density and density of both intensively and extensively raised chickens were also found to be predictors of H7N9 presence^26^. A previous study also found there was a major shift of risk factors from anthropological (i.e. LBM and human population density) towards poultry related variables (i.e. poultry density and chicken-to-duck ratio) linked to human H7N9 cases over time^3^. Other studies also evaluated the role of pig density, distance to freeway, distance to national highway, landcover, temperature and relative humidity, etc.^22,25^. However, none of these studies looked at the effect of more proximal factors such as poultry surveillance results and live chicken movement in explaining the geographical variation of human H7N9 infections.

In this study, we quantified the temporal relationship between the onset of human H7N9 infections during 2013–2017 and poultry serological and virological surveillance results. Then we estimated the relative risk of H7N9 human incidence in counties in Southeast China by assessing the relationship between human infections as the outcome and poultry virological surveillance results, live chicken movements and recognized demographic risk factors as explanatory variables.

## Results

**Figure 1:**
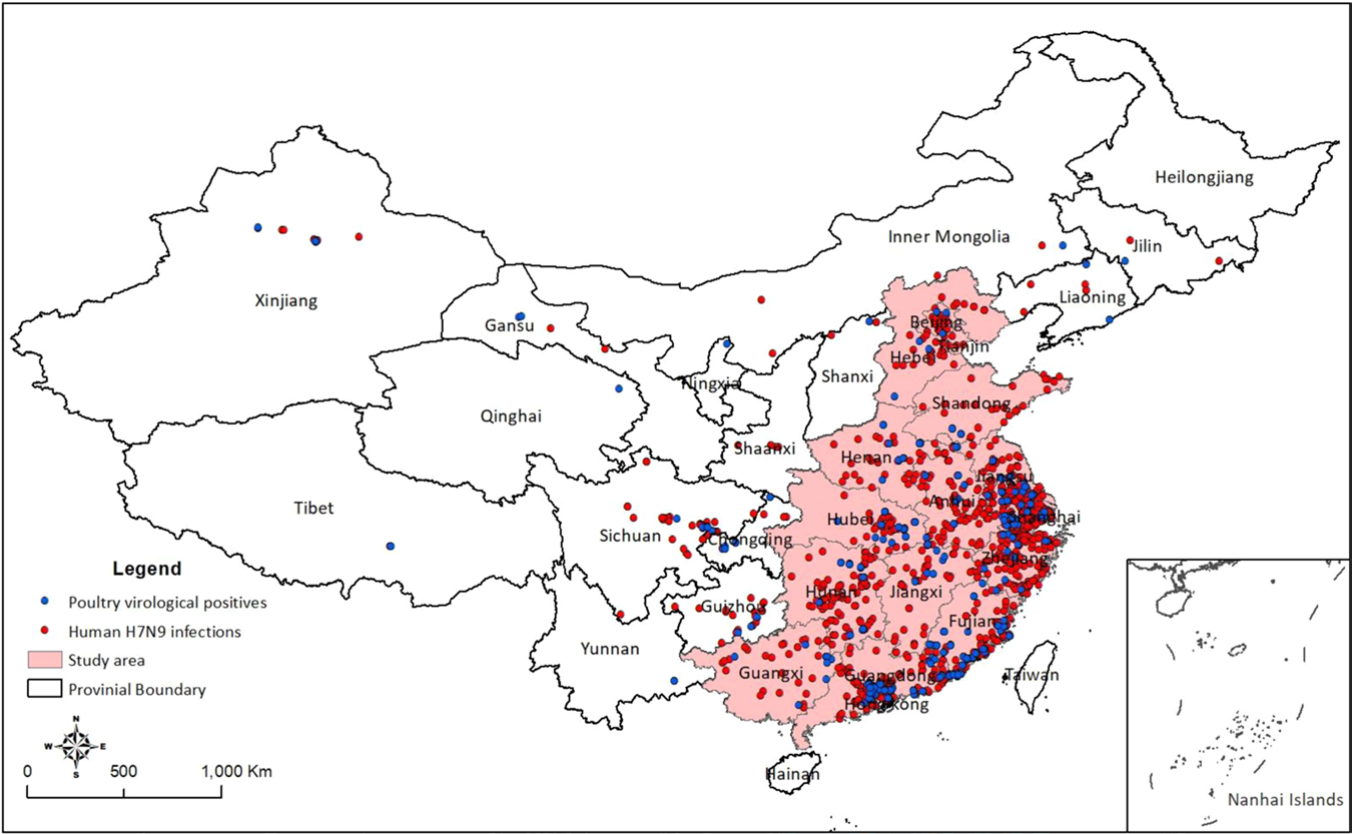
Spatial distribution of human H7N9 infections (red dots) and poultry virological surveillance positives (blue dots) from 2013 to 2017. Dots represent either geographic locations of the H7N9 human infections or county centroids when the detailed location was not available.

The distribution of human H7N9 notifications and poultry surveillance positives from Mar 2013- Aug 2017 is shown in Fig. 1. A total of 1,514 human H7N9 infections and 324 poultry virological positives were geocoded at least to county level. The majority of the H7N9 virological positive samples (88.3%, 286 out of 324) were collected from LBMs. A total of 1,181 counties from 14 provinces and municipalities in southeast China were included in this study (Fig. 1); about 93% (1408 out of 1514) of reported human H7N9 infections and 89.5% (290 out of 324) of reported H7N9 virological positive samples fell in these counties.

### Social network analysis of chicken movements

In total, we analyzed live chicken movement data from four wholesale LBMs and four live poultry trading platforms from Jiangsu, Anhui and Shanghai from January to July 2014 (Table S1). Chicken movements from live poultry trading platforms tend to involve long-distance and inter-provincial transportation of chickens, while chicken movements from wholesale LBMs are mostly confined to local areas or neighboring provinces (Fig. S1). The full extent of the 2-mode network (LBMs and chicken source/destination counties) is presented in Supplementary Fig. S2. The results of this analysis revealed that there was a giant weakly connected component comprising eight wholesale LBMs and 249 chicken source/destination counties. These 249 counties were located mainly in Jiangsu, Anhui and Shanghai, extending to neighboring provinces Henan, Hubei and Shandong, and further to the south, including Guangdong. The degree centralities of all the county nodes ranged from one to six and the geographic distribution of degree centrality is demonstrated in Fig. S3. The counties with the highest degree centrality were Jintan, Changzhou, Yangzhou in Jiangsu province (degree = 6); and Jiangyan, Lishui, Taixin, Shuyang, Zhenjiang and Nanjing from Jiangsu province, and Huzhou from Zhejiang province and Wuhu from Anhui province (degree = 5).

### Temporal associations between human H7N9 notifications and poultry H7N9 surveillance data

**Figure 2:**
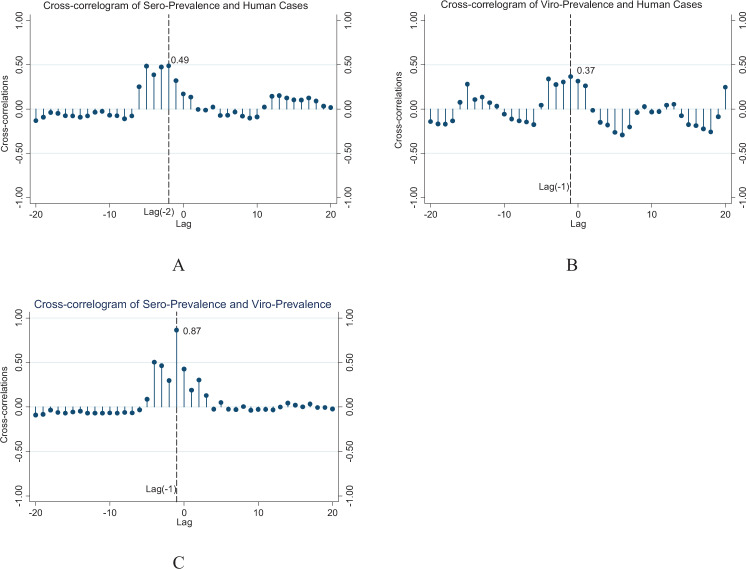
Cross correlation of H7N9 poultry serological prevalence and virological prevalence relate to H7N9 human cases (**A**,**B**), and cross correlation of H7N9 serological prevalence relate to virological prevalence (**C**). The correlation of sero-prevalence at Lag −2 is approximately 0.49, and the correlation of viro-prevalence at Lag −1 is approximately 0.37. The correlations are significant because the values are greater than [2/ (Sqrt (n-|lag | ))), n = 48].

Results from the time-series analysis indicate that there is a significant temporal relationship between human H7N9 notifications and poultry surveillance results. Our results indicate that the peak of poultry H7N9 serological positives is followed by human H7N9 infections with a two-month lag, poultry H7N9 virological positives are followed by human H7N9 infections with a one-month lag (Fig. 2). In addition, poultry serological H7N9 positives are followed by poultry H7N9 virological positives with a one-month lag (Fig. 2).

### Spatial autocorrelation (Moran’s I)

Incidence of human H7N9 infections was significantly spatially clustered, as indicated by a positive Moran’s I value (0.152) that was statistically significant at the 0.05 level (Table S2).

### Bayesian spatial conditional autoregressive model of human H7N9 infections

The presence of wholesale LBMs (Coef. = 0.33, 95% CrI: 0.08~0.56) in the county and the density of retail markets (Coef. = 0.88, 95% CrI: 0.52~1.24) were positively and significantly associated with the human H7N9 incidence (Table 1). Human H7N9 incidence was positively associated with the presence of poultry virological positives (Coef. = 0.56, 95% CrI: 0.27~0.84) and the connectivity of counties with respect to poultry movements (Coef. = 0.81, 95% CrI: 0.46~1.18; Coef. = 0.89, 95% CrI: 0.23~1.58). While human H7N9 incidence was positively associated with increasing chicken population density (Coef. = 0.33, 95% CrI: 0.01~0.65; Coef. = 0.91, 95% CrI: 0.44~1.38), human H7N9 incidence was inversely proportional to human population density (Coef. = −0.70, 95% CrI: −1.00~−0.40; Coef. = −1.12, 95% CrI: −1.50~−0.74).

**Table 1:** Results of spatial conditional autoregressive model of human H7N9 human incidence during 2013–2017.

Variables at county level	Category	Coefficient, posterior mean (95%CrI)
Present of wholesale LBMs	no	Ref.
	yes	**0.33 (0.08~0.56)**
Retail LBMs density (markets/100 km^2^)	Low density (<1)	Ref.
	Medium density (1–3)	0.16 (−0.13~0.44)
	High density (>3)	**0.88 (0.52~1.24)**
Present of poultry virological positive	no	Ref.
	yes	**0.56 (0.27~0.84)**
Population density (people/km^2^)	0–200	Ref.
	201–600	**−0.70 (−1.00~−0.40)**
	>600	**−1.12 (−1.50~−0.74)**
Chicken density (birds/km^2^)	<500	Ref.
	500–3000	**0.33 (0.01~0.65)**
	>3000	**0.91 (0.44~1.38)**
Network estimate (degree centrality)	0	Ref.
	1~3	**0.81 (0.46~1.18)**
	4~6	**0.89 (0.23~1.58)**
Intercept		−1.46 (−1.80~−1.13)
Precision of spatial random effect		0.22 (0.18~0.27)

(CrI Credible Interval, a variable was considered significant if CrI excluded 0).

**Figure 3:**
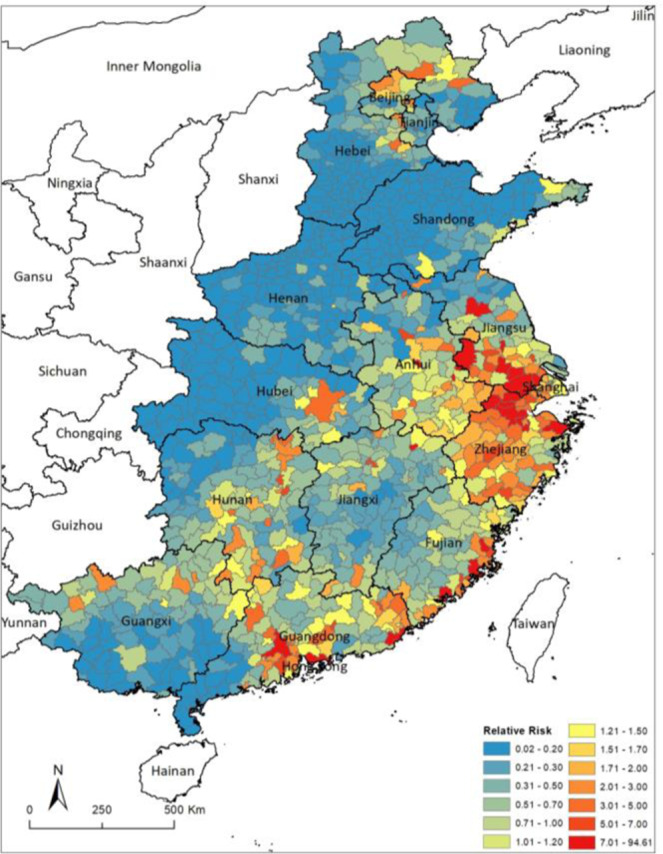
Spatial distribution of the relative risks for human H7N9 incidence in counties in southeast provinces. Red and bright color indicating a higher risk, blue and darker color indicating a lower risk. The maps were created in ArcGIS 10.1 software (©ESRI).

A map of adjusted relative risks (RRs) of human H7N9 incidence by county (Fig. 3) shows that high risk areas of human H7N9 infection were spatially clustered in southeastern China, extending from the Yangtze River delta near Shanghai to the Pearl River delta near Guangzhou and covering most areas of Jiangsu, Zhejiang, Shanghai, Anhui, Fujian, Guangdong and Hunan provinces. Additional hot spots for human H7N9 infections were found in the northern region of Guangxi, eastern region of Hubei province, northern and southern region of Jiangxi, northern region of Beijing, and the northern region of Hebei province (Fig. 3). The map of the spatially structured random effects demonstrates evidence of clustering around the Yangtze River delta area (Fig. S4).

## Discussion

This study extends current knowledge^3,22–26^ about the spatiotemporal epidemiology of human H7N9 infections in a number of ways. Firstly, using the most complete data on human H7N9 infections and poultry LBM surveillance from 2013–2017, our spatial analyses mapped the spatial distribution of human H7N9 infections and its relationship with poultry serological and virological surveillance results. Second, our human H7N9 relative risk map displayed the distribution of high-risk areas associated with poultry infection status in the county, presence of wholesale LBMs, density of retail LBMs, human population density, chicken density and poultry movement network in the county.

Our analysis identified temporal lags between human H7N9 notifications and poultry surveillance recorded during 2013 to 2017. From examining the temporal relationship between human H7N9 infections and poultry H7N9 surveillance results, we detected a one/two-month temporal lag between the onset of human H7N9 infections and poultry virological/serological surveillance results. These temporal lags may be explained by, firstly, the sensitivity of serological surveillance for H7N9 in poultry is much higher than virological surveillance, and LPAI virus or its genome can be detected in an individual bird for only a few days due to the short period of virus shedding, whereas antibodies elicited by LPAI virus are often present for the entire production life of the infected poultry^27,28^. Meanwhile, due to low sensitivity, virological positives will be more likely to be detected when the concentration of virus has built up to a more detectable level most likely through the live poultry market chain, i.e. from farms then going through traders, wholesale markets and retail markets. Besides, our results demonstrated that most of the H7N9 virological positive samples were collected in LBMs^10^, which is consistent with the consensus that the primary risk factor for human H7N9 infections in China is exposure to LBMs^11–17^. These findings are also consistent with those from our spatial models of human H7N9 incidence, suggesting that the county-level incidence of human H7N9 infections is positively associated with the presence of poultry virological positives in the county. Together these findings have important operational implications for anticipating human H7N9 infections based on current routine LBM H7N9 surveillance in poultry.

Previous studies indicated that LBM density and the number of LBMs were important factors for explaining the risk of H7N9 human infections^3,13,22,23,25,26,29^. In our analysis, both the presence of wholesale LBMs and density of retail LBMs were positively associated with higher relative risk of human H7N9 infections. Wholesale LBMs bring together live birds from large catchment areas and birds are commonly traded to retail LBMs^30,31^; this results in market networks with numerous trade connections. Higher densities of markets may exacerbate that risk and explain the strong spatial correlation with suitability for H7N9 infection^26^. Closing LBMs appears to be an effective approach for eradicating or reducing H7N9 infections in humans^32^. However, a recent study presented evidence that the closure of LBMs in early waves of H7N9 influenza had resulted in expansion of H7N9 infection to uninfected areas^20^. This implies closing LBMs is a long-term strategy that needs to be further evaluated. Our recent meta-analysis identified biosecurity measures that have been effective for controlling AI viruses at LBMs include smaller market size, selling single poultry species and separating different species, mandatory monthly rest days and bans on keeping live birds overnight, and sourcing poultry from local areas^33^. These identified characteristics of LBMs allow us to better target control efforts.

Furthermore, in our model we included estimates of live chicken movement from areas originally affected areas by H7N9 in Southeast China, which allowed us to evaluate the effect of live chicken movement from the primary high-risk area on the overall distribution of human H7N9 infections from 2013 to 2017. Our results indicate a positive relationship between human H7N9 incidence and poultry movement estimates (degree centrality) from our CAR model. A previous study of poultry market chains in South China also reported that LBMs where HPAIV H5N1 was isolated were associated with higher degree centrality^34^. Poultry network studies in Vietnam and South China revealed that live poultry traders tend to link poultry sources of similar infection status^34,35^. These findings suggest that poultry movements from the originally affected area in east China provinces may continue to play a role in disseminating H7N9 virus throughout China. This further demonstrates the importance of evaluating live poultry movement and trading practices to develop appropriate and targeted surveillance recommendations for active H7N9 surveillance program.

After adjusting for poultry marketing system variables (presence of wholesale LBMs and density of retail LBMs) and spatial autocorrelation, our results indicated that human population density was negatively associated with the human H7N9 incidence while chicken density was positively associated with human H7N9 incidence. This can partly be explained by the known epidemiology of H7N9 in humans in that most human cases are a result of animal-to-human transmission, rather than human-to-human transmission. Since most H7N9 cases have been reported in large cities where human population density is very high, it may partially due to that the surveillance effort to detect H7N9 human cases was much greater in area with high population density and better medical facilities^22,23^. Moreover, higher human population density is usually related to higher biosecurity levels in the LBMs in highly dense urbanized areas. Furthermore, existing evidence indicates that H7N9 is more prevalent in chickens than in other poultry species^3,8,36^. Also, while H7N9 can affect other species it is mainly limited to chickens due the characteristics of the industry and the marketing system^8^. Higher chicken density is usually related to high chicken production, chicken trading and transportation which may promote transmission of the pathogen among poultry and increase the chance of humans acquiring H7N9 infection. Our findings suggest that highly connected areas with high chicken density and low human population should be targeted in case the virus continues to evolve or the efficacy of the vaccine is reduced, or even for the emergence of similar viruses in the future.

Moreover, the results of our study demonstrated significant spatial clustering of human H7N9 incidence in the study area, which required the development of a geographical model that incorporated spatial autocorrelation in order to generate a robust risk map of human H7N9 infection across China. Our human H7N9 relative risk map suggests that although H7N9 vaccine for poultry is currently available, continued active surveillance still needs to be strengthened for high-risk areas in China. Our results support strengthening LBM and human surveillance in Southeast area of China (involving Jiangsu, Zhejiang, Anhui provinces and Shanghai Municipality), coastal areas in Fujian and Guangdong provinces, and some inland areas in Hubei, Hunan and Guangxi provinces, as well as Beijing Municipality and the Northern area in Hebei province. According to the National Guidelines on the Prevention and Control of H7N9 influenza in Poultry in China (2018–2020)^37^, the current control of H7N9 infections in poultry in China has relied heavily on wide-scale compulsory preventive vaccination combined with biosecurity enhancement in both poultry farms and LBMs, regular surveillance programs, as well as live poultry movement control, quarantine and stamping out. The introduction of live poultry from high-risk areas and sites is strictly restricted^37^, however, the delimitation of high risk-areas is unclear. This study attempted a new risk assessment approach and the results provided recommendations to a more targeted risk-based surveillance program, as well as new insights into the role of LBMs and poultry movement in China. However, the map of the spatially structured random effects demonstrates evidence of clustering around the Yangtze River delta area, suggesting that there are other risk factors not included in our spatial models, such as people’s behaviour, or indeed other environmental factors that could account for the residual spatial distribution.

The results of this study should be interpreted in light of some limitations. Our analyses were based on laboratory-confirmed cases of human H7N9 infections and reported poultry H7N9 virological surveillance results, and are therefore subject to reporting bias, especially in areas of China with poor surveillance system coverage. In addition, our data for the distribution of LBMs were obtained from local veterinary departments except Shandong and Zhejiang provinces, data for these two provinces were replaced by another dataset clarified in the supplementary file, which may bring some reporting bias and uncertainty to the model. Furthermore, our live chicken movement data were collected in selected high-risk areas in Southeast provinces in 2014, representing the live chicken movements coming from and to the originally affected provinces, which may not reflect the current poultry movement situation across the region. The measures of degree centrality used in our model do not represent a perfect indicator of the “overtime” exposure of countries via movement of poultry from 2013 throughout 2017, its use in our model is important to consider in the context of the original source of the virus. We recognize that the effect of the measures of degree centrality are far from depicting a causal relationship and thus are prone to regression dilution bias; however, it is remarkable that despite this limitation we were able to identify a significant signal on the role of the poultry movements originating from the initially affected area.

In conclusion, contamination of LBMs with H7N9 is an important determinant of the risk of human H7N9 incidence in China. Moreover, poultry movement from the original areas of H7N9 emergence may be an important driver of the dissemination of H7N9 infections across China, and poultry serological positives and virological positives can serve as a predictor for human H7N9 infections as well as being a guide for the timing of risk management interventions. Highly connected areas with high chicken density and low human population should be targeted. It is recommended that regular monitoring of poultry movement and poultry infections at the high-risk counties identified in this study will provide essential evidence for the early warning of H7N9 infections across China.

## Materials and Methods

### Human H7N9 infection data and poultry H7N9 surveillance data

We obtained all laboratory-confirmed H7N9 human cases reported during Mar 2013 and Aug 2017, from “Situation Updates - Avian Influenza” of the World Health Organization (WHO)^38^ and “Avian Influenza Report” from the Centre for Health Protection of the Department of Health of the Hong Kong Special Administrative Region (SAR)^39^. Case definitions and laboratory testing have been described previously^22,40^. For each human H7N9 case, information on county of residence and date of onset of symptoms was extracted.

For poultry H7N9 infection data, we extracted data on H7N9 poultry surveillance between Mar 2013 and Aug 2017 from the National H7N9 Surveillance Program. Both poultry serological and virological surveillance results are published on the monthly official Veterinary Bulletin released by the Veterinary Bureau of the MARA of China^10^. Since April 2013 Chinese authorities conduct routine active surveillance for H7N9 in poultry throughout the territory^41^. The National H7N9 Surveillance Program is an active surveillance system that consists of monthly serological and virological surveillance carried out by provincial Centers for Animal Disease Control and Prevention (CADCs). The samples were collected by county level CADCs, then the samples will be gathered by prefecture level CADCs. Identification of H7N9 infection in poultry involves testing of oropharyngeal and cloacal swab samples using RT-PCR or fluorescence RT-PCR and positive results are confirmed by the Harbin National Avian Influenza Research Institute. Serological tests against H7 are used to determine whether poultry were exposed to an H7 virus, serum samples are tested by hemagglutination inhibition (HI) test. The surveillance targets are specified chickens (especially layer, yellow feather broilers and other breeds which have long raising cycle), waterfowl (ducks, geese), domestic pigeons and quail, wild birds and environment in high risk areas. The scope of surveillance was specified to be all poultry trading markets in China, stalls selling live poultry in farmers markets, poultry with certain size, backyard poultry raising farmers, poultry slaughter houses, and habitats of migratory birds. Sample collection was done through the national surveillance program performed by provincial animal CDCs on a monthly basis. All reported H7N9 human cases and poultry virological surveillance positives were then geo-referenced and linked to a county level map of China.

### Data on live chicken movement

A cross-sectional survey was conducted from June to July in 2014 targeting the live meat chicken trade in six counties located in Jiangsu (Lishui, Jintan, Jiangyan) and Anhui (Feixi, Quanjiao and Chaohu) provinces. These counties were selected based on the findings from a previous study^8^ conducted during the H7N9 emergency response, which demonstrated that connectivity of chicken sources was highest in these six counties. In addition, one county in Shanghai municipality (Fengxian) was added, which was the location of the first H7N9 human case reported in 2013. Shanghai municipality is also adjacent to Jiangsu province where human H7N9 and H5N1 infections were demonstrated to be co-distributed^42^. An initial sample size of one or two wholesale LBMs (subject to actual numbers of LBMs in a county) in the seven high-risk counties were included; typically, there are a total of 1 to 2 wholesale LBMs in each county. Information on live poultry movements was obtained from poultry movement certificates available at wholesale LBMs and live poultry trading platforms in the selected counties. Live poultry trading platforms are intermediary poultry trade venues that operate between wholesalers and farmers. For instance, a wholesaler places an order at the trading platform for 5,000 meat chicken and the trading platform will contact farmers who raise meat chickens. Farmers with meat chicken in stock will then deliver poultry to the trading platform and the wholesaler poultry trader will then purchase the chicken at the trading platform and move them to the wholesale LBM. All available chicken movement certificates in the trading locations surveyed were recorded from January 2014 to July 2014. This dataset records half-year chicken movements and is representative of the extent of live chicken movement within and beyond that region.

### Sociodemographic factors

A set of sociodemographic risk factors was considered in the analysis including the presence of wholesale LBMs in each county (a binary variable representing presence/absence), and the density of retail LBMs (markets/100 km^2^), human population density (people/km^2^) and chicken density (birds/km^2^). All factors were compiled at the county level. All data sources are summarized in Table 2; the source of LBMs is described in the Supplementary Information 1.

**Table 2:** Risk factor variables used in the analysis.

Variables at county level	Sources
Presence of wholesale LBMs	China Animal Health and Epidemiology Centre (see supplementary information)
Number of retail LBMs	China Animal Health and Epidemiology Centre (see supplementary information)
Poultry virological positives	Monthly Veterinary Bulletin from MARA
Network centrality	Primary investigation in Jun-Jul 2014
Human population density	2010 Census
Chicken density	Robinson *et al*. 2007^47^

### Social network analysis

To describe the connectivity pattern within the chicken movement dataset consisting of records of paired trading events between a particular LBM and the county they trade with (termed as “trade county”), we used social network analysis (SNA), as described previously^34^. We summarized network connectivity using degree centrality of the 2-mode binary network (LBM nodes vs trade county nodes). The degree represents the absolute number of unique links of a given node and it is important for describing the levels of connectivity between different actors within a network, thereby allowing identification of the most influential spreaders within a network^43^.

### Cross correlation analysis

To assess the temporal relationship between the onset of human H7N9 infections and poultry serological and virological surveillance results, we used a time-series cross-correlation analysis to calculate the temporal lags in months between the outcome (human infections) and the surveillance indicator (poultry infections). The dataset was structured by month because the reports of surveillance results were aggregated by month^10^. In order to mitigate potential missing detection and report in early 2013 and potential reporting bias in late 2017 after the adoption of H7 vaccination since July 2017, we only used human and poultry H7N9 infection data from July 2013 to June 2017. From each time-lagged correlation, only the lag with the highest correlation value was selected for the analysis. Usually, a correlation is significant when the absolute value is greater than 2/sqrt(n-|k | ), where n is the number of observations and k is the lag.

### Analysis of spatial variation in human H7N9 infections at county level

To assess whether there was spatial autocorrelation in the observed pattern of human H7N9 infections in the study area we used the Global Moran’s Index (Moran’s I), a measure of spatial autocorrelation for spatially aggregated data. We used the incidence rate of human H7N9 infections per 1,000 (i.e. estimated by dividing the observed number of human cases by the total human population in the county and multiplied by 1,000) for estimation of Moran’s I. Moran’s I is positive when nearby areas tend to be similar, negative when they tend to be dissimilar, and approximately zero when attribute values are arranged randomly in space^44^. The Moran’s I value and a Z-score (evaluating the significance of the index) were estimated using ArcGIS 10.1 (©ESRI).

### Bayesian spatial conditional autoregressive model (CAR)

A Bayesian framework was used to construct a Poisson regression model of the observed incidence of human H7N9 infections in each county using the OpenBUGS software 3.2.3 rev 1012^45^. The model included all of the explanatory variables described above and a spatially structured random effect. The mathematical notation for the model is provided in the Supplementary Information 2. It assumed that the observed counts of H7N9 human infections in the county (from 1 to 1181) followed a Poisson distribution.

The spatially structured random effect was modelled using a conditional autoregressive (CAR) prior structure^46^. This approach uses an adjacency weights matrix to determine spatial relationships between counties. If two counties share a border, it was assumed the weight = 1 and if they do not the weight = 0. The adjacency matrix was constructed using the “Adjacency for WinBUGS tools” in ArcGIS^45^. A flat prior distribution was specified for the intercept, whereas a normal non-informative prior distribution was used for the coefficients (with a mean = 0 and a precision = 0.001). The priors for the precision of spatially structured random effects were specified using non-informative gamma distributions (0.5, 0.0005). The OpenBugs code is in Supplementary Information 3.

The first 1,000 iterations were run as a burn-in period and discarded. Subsequent sets of 20,000 iterations were run and examined for convergence. Convergence was determined by visual inspection of posterior density and history plots and by examining autocorrelation plots of model parameters. Convergence occurred at approximately 100,000 iterations for each model. Another 20,000 values from the posterior distributions of the model parameters were stored and summarized for the analysis. Statistical significance was indicated by 95% credible intervals (95% CrI), a variable was considered significant if CrI excluded 0.

Choropleth maps were created using the ArcGIS software to visualize the geographical distribution of crude incidence for the 1181 counties in the study area. The posterior means of the CAR random effects obtained from the models were also mapped.

### Ethics statement

The research proposal leading to the study received ethical approval from the China Animal Health and Epidemiology Centre (CAHEC) of MARA China. The research proposal leading to the primary data collection of chicken movements received ethics approval from the Behavioral & Social Sciences Ethical Review Committee of the University of Queensland (Approval number: 2014001167). There were no samples from humans or animals taken as part of our study, and we used secondary information on human infections and market positivity to H7N9 infection derived from open access websites. All methods were performed in accordance with the relevant guidelines and regulations.

## Supplemenatry information

Supplemenatry information.
