# Supplementary material for: Geographical variation in the risk of H7N9 human infections in China: implications for risk-based surveillance
Source: Sci Rep. 2020 Jun 25;10:10372. doi: 10.1038/s41598-020-66359-1 (PMC7316858; doi:10.1038/s41598-020-66359-1)
Supplement: Supplementary file 1 — Supplemenatry information. [file 41598_2020_66359_MOESM1_ESM.docx]

**Geographical variation in the risk of H7N9 human infections in China: implications for risk-based surveillance**

**Authors:** Xiaoyan Zhou^1^, Lu Gao^2^, Youming Wang^2^, Yin Li^2, 3^, Yi Zhang^2^, Chaojian Shen^2^, Ailing Liu^2^, Qi Yu^4^, Wenyi Zhang^5^, Alexander Pekin^1^, Fusheng Guo^6^, Carl Smith^7^, Archie C.A. Clements^8, 9^, John Edwards^1, 2, 3^, Baoxu Huang^2^, Ricardo J. Soares Magalhães^1, 10^

**Affiliations:**

^1^ School of Veterinary Science, The University of Queensland, Australia

^2^ China Animal Health and Epidemiology Centre, Ministry of Agriculture, PR China

^3^ School of Veterinary and Biomedical Sciences, Murdoch University, Australia

^4^ Beijing Center for Animal Disease Prevention and Control, Beijing, PR China

^5^ Institute of Disease Control and Prevention, Academy of Military Medical Science, Beijing, PR China

^6^ Food and Agriculture Organization of the United Nations (FAO), Bangkok

^7^ School of Business, the University of Queensland, Australia

^8^ Faculty of Health Sciences, Curtin University, Australia

^9^ Telethon Kids Institute, Australia

^10^ UQ Child Health Research Centre, The University of Queensland, Australia

**Corresponding Author:**

Baoxu Huang, [huangbx@cahec.cn](mailto:huangbx@cahec.cn), China Animal Health and Epidemiology Centre, Qingdao, Shandong, PR China

Xiaoyan Zhou, [xiaoyan.zhou@uq.edu.au](mailto:xiaoyan.zhou@uq.edu.au), [zhouxy339@outlook.com](mailto:zhouxy339@outlook.com), University of Queensland, Gatton, QLD 4343, Australia

**Supplementary Figures**


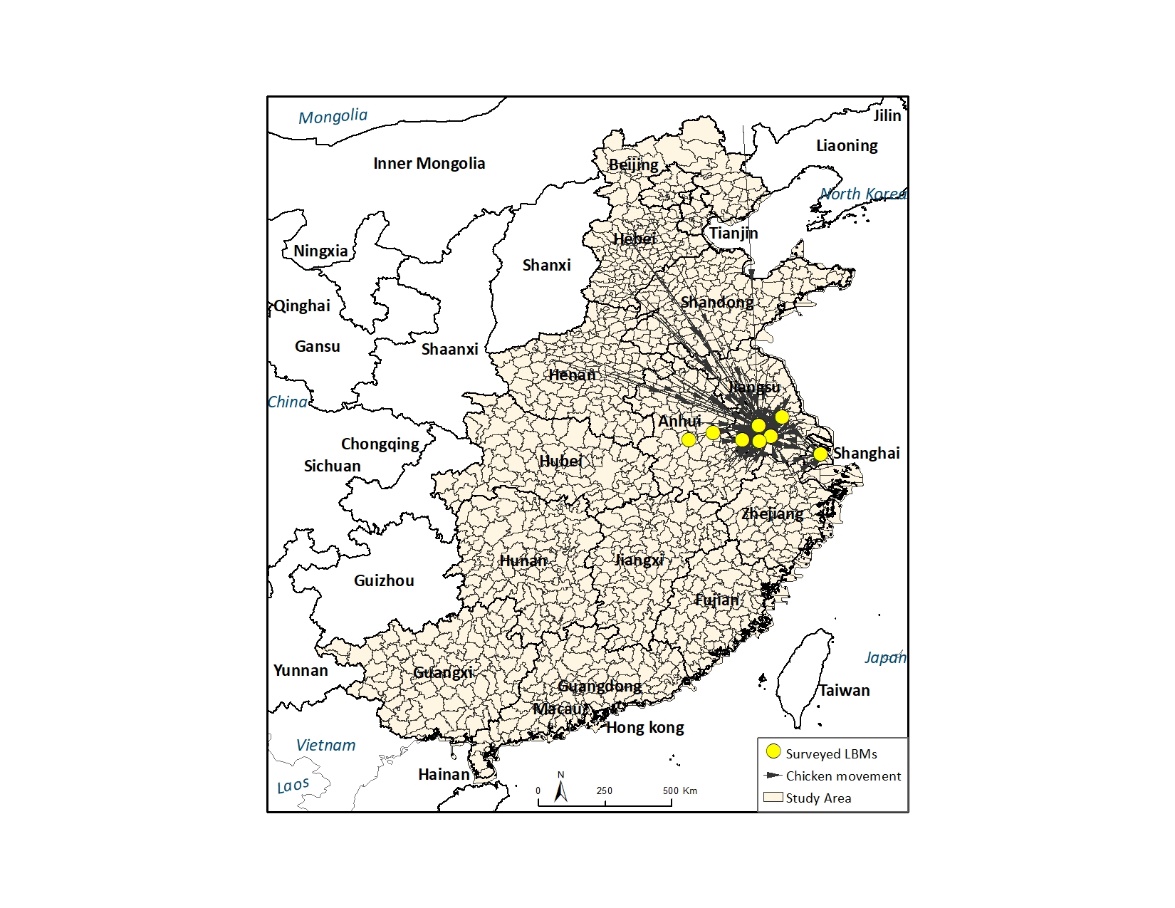

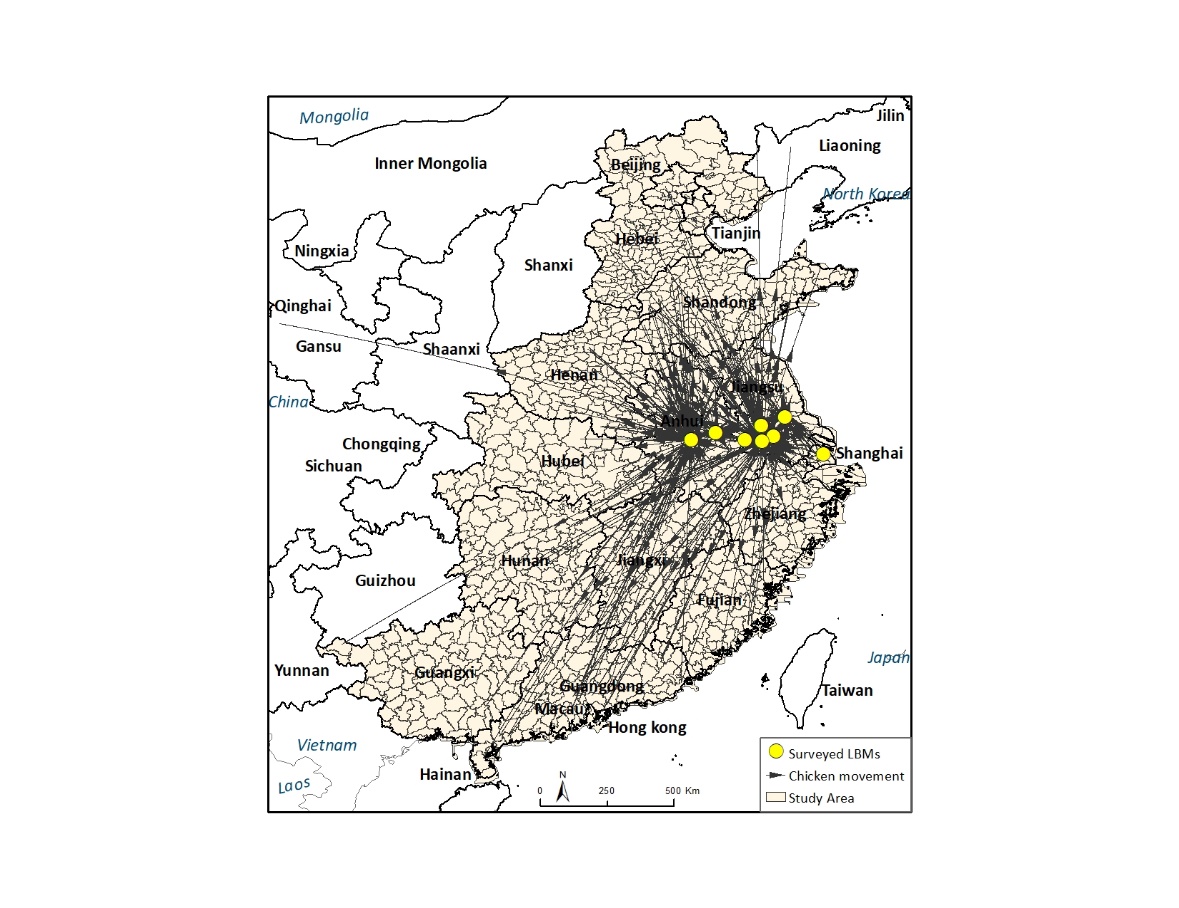


**Figure S1.** Geographic distribution of live chicken movements from wholesale LBMs (left) and live poultry trading platforms (right).


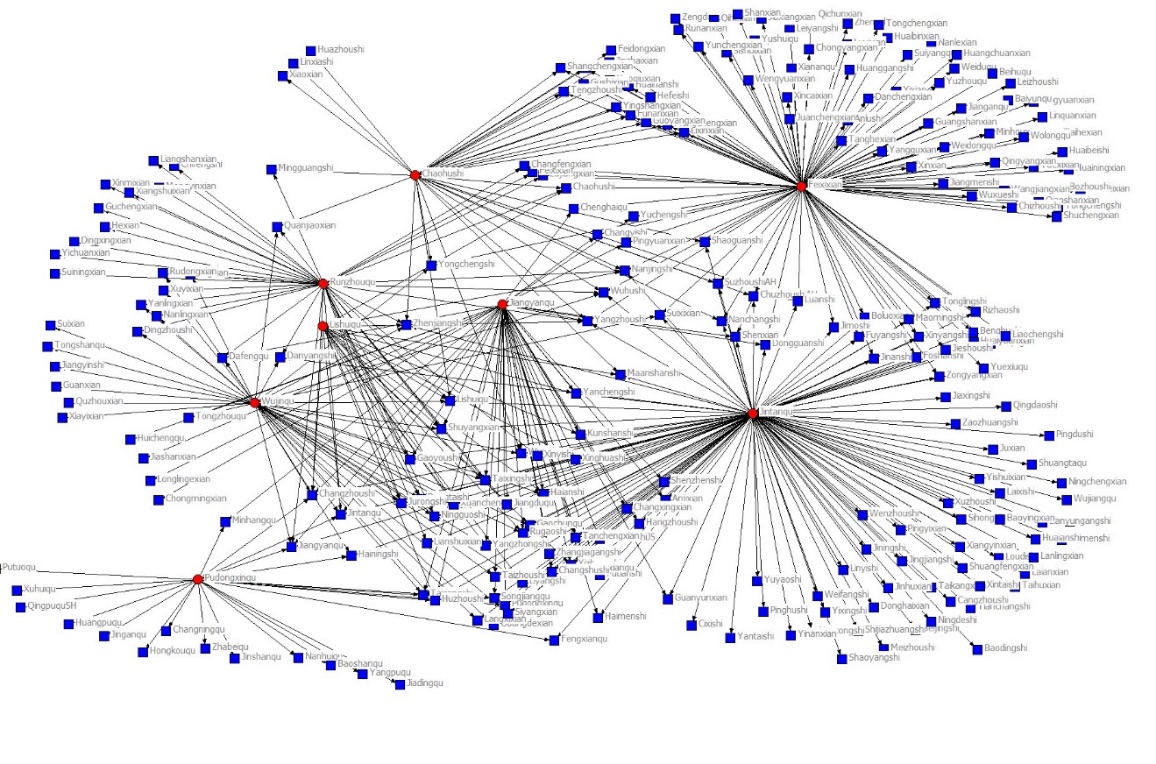


**Figure S2.** 2-mode Network between surveyed LBMs/poultry trading platforms and counties of live chicken sources/destinations.


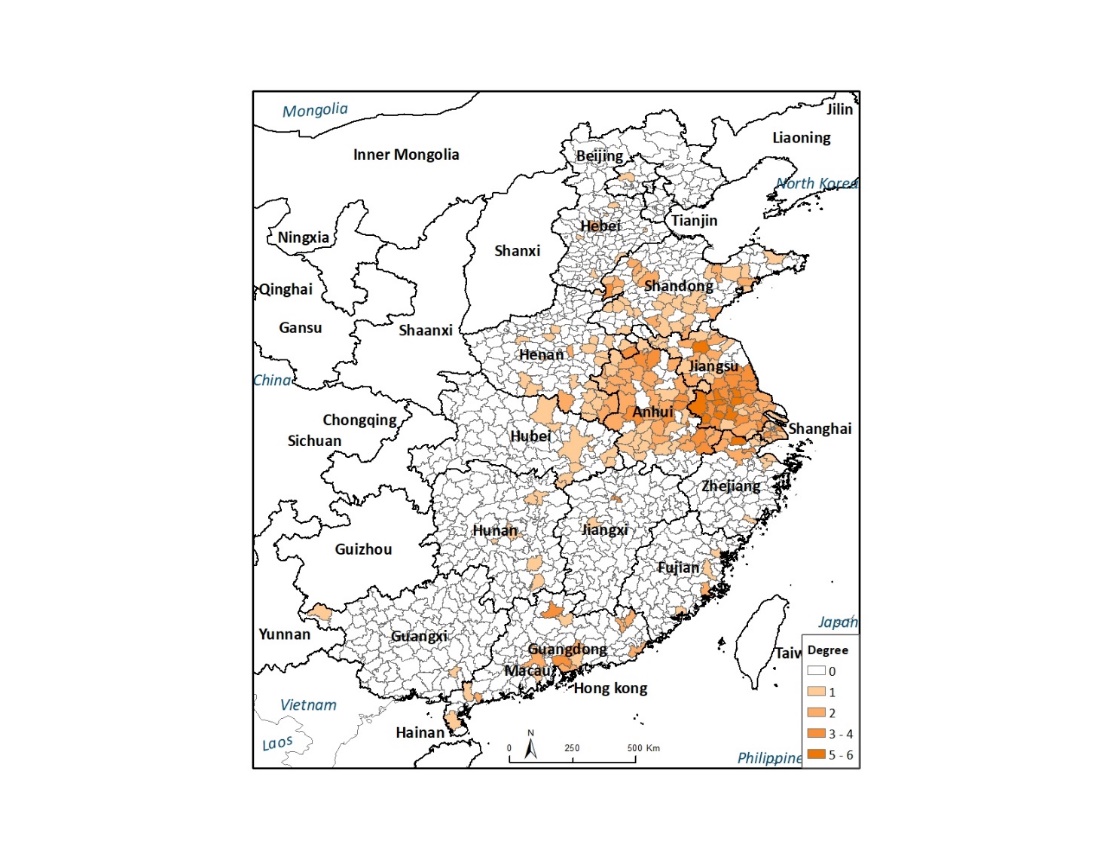


**Figure S3.** Geographical distribution of the degree centrality of live chicken sources/destinations (county level), based on a 2-mode network of live chicken movements.


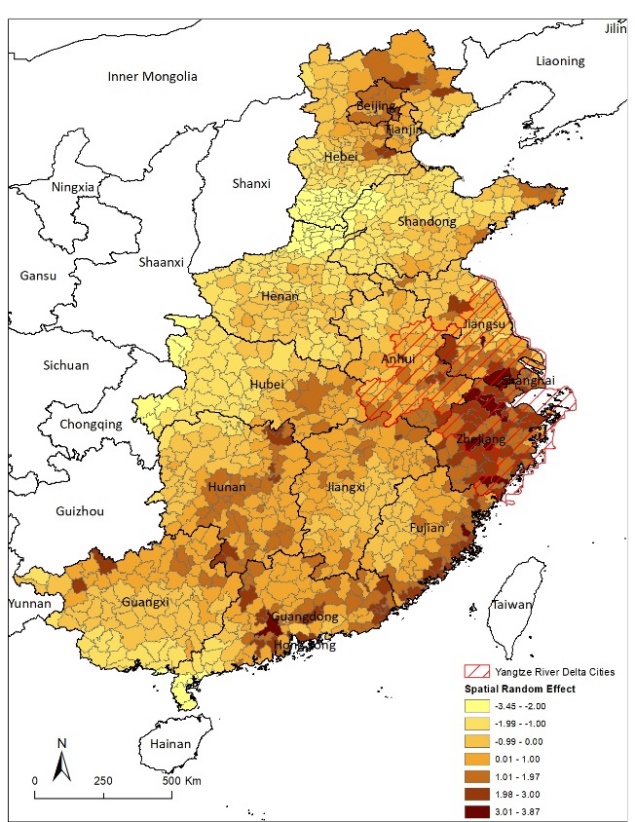


**Figure S4.** Spatial distribution of spatially structured random effects of the CAR model for human H7N9 infections. The maps were created in ArcGIS 10.1 software (ESRI Inc., Redlands, CA, USA) (<http://www.esri.com/>). The area of Yangtze River Delta cities is mapped based on the ‘Outline of the integrated regional development of the Yangtze River Delta’ jointly issued by the Communist Party of China Central Committee and the State Council ^1^.

**Supplementary Tables**

**Table S1.** List of surveyed sites of chicken movements.

| No. | Province | Records From | NameEN | Start Date | End Date | Incoming  records | Outgoing  records | Degree |
| --- | --- | --- | --- | --- | --- | --- | --- | --- |
| 1 | Shanghai | Wholesale LBM | Shanghai Nongpi | 1/01/2014 | 16/07/2014 | 1247 | 6061 | 23 |
| 2 | Jiangsu | Wholesale LBM | Changzhou Lingjiatang | 1/01/2014 | 24/07/2014 | 2540 | * | 43 |
| 3 | Jiangsu | Wholesale LBM | Zhenjiang Nongfuchanpin | 9/01/2014 | 24/07/2014 | 1674 | * | 42 |
| 4 | Jiangsu | Wholesale LBM | Lishui Wenshi | 1/01/2014 | 22/07/2014 | 715 | * | 24 |
| 5 | Jiangsu | Trading platform | Changzhou Tianmu Lihua | 1/1/2014 | 30/06/2014 | * | 8693 | 124 |
| 6 | Jiangsu | Trading platform | Jiangyan Heyin | 2/02/2014 | 25/07/2014 | * | 703 | 46 |
| 7 | Anhui | Trading platform | Chaohu Zhengkang | 1/01/2014 | 16/06/2014 | * | 323 | 33 |
| 8 | Anhui | Trading platform | Feixi Wenshi | 1/01/2014 | 20/06/2014 | * | 1383 | 105 |

**Table S2.** Summary of Moran’s I index of incidence rate of human H7N9 infections.

|  | Number of observed human H7N9 infections |
| --- | --- |
| Moran's Index: | 0.1525 |
| Expected Index: | -0.00085 |
| Variance: | 0.00018 |
| z-score: | 11.3533 |
| p-value: | 0.0000 |

**Supplementary Information 1: Data source of wholesale LBMs and retail LBMs**

The presence of wholesale LBMs and number of retail LBMs were obtained from a survey conducted by China Animal Health and Epidemiology Centre (CAHEC). The provincial Center of Animal Diseases Control and Prevention (CADC) were approached by CAHEC to provide with number of wholesale and retail LBMs in each county in the province. However, LBMs data in Zhejiang and Shandong provinces were missing. Therefore, we used the Points of Interest (POI) data from year 2012 for these two provinces as a database, and we searched with different combinations of terms, i.e., live bird markets, famers’ markets, agricultural products markets and wet markets. We then carefully screened each market name and make sure all included markets are agriculture product related markets. We used this dataset as a substitute to live bird markets in the two provinces.

**Supplementary Information 2: Mathematical notation for the Bayesian spatial CAR model**

It assumed that the observed counts of the H7N9 human infection, for the ith county (i = 1 to 1181) followed a Poisson distribution with mean (μij), that is,

Y_i_ ∼ Poisson(μ_i_)

log(μ_i_) = log (Exp_i_) + θ_i_

θi = α + x * γ + ∑β_z_ * λ_zi_ + s_i_

where Exp_i_is the expected number of human H7N9 cases in county i (acting as an offset to control for population size) and θ*_i_* is the mean log relative risk (RR); α is the intercept, γ is the coefficient for temporal trend, β is a vector of z coefficients, λ is a matrix of z environmental covariates, and s_i_is the spatially structured random effect with mean zero and variance σ_s_^2^. Standardization of environmental variables was used to allow comparability of the effects and provide a more meaningful interpretation on the results.

**Supplementary Information 3:** OpenBUGS code used to develop the Bayesian spatial model for H7N9 human infections from 2013 to 2017.

model {

#CAR prior distribution for spatial random effects:

s[1:1181] ~ car.normal(adj[], weights[], num[], tau.s)

for(k in 1:sumNumNeigh) {

weights[k] <- 1

}

for (i in 1:1181) {

O[i] ~ dpois(mu[i])

log(mu[i]) <- log(E[i]) + log.RR[i]

log.RR[i] <- alpha + U[i] + s[i]

U[i] <- beta1 * WsM[i] + beta2 * ReMDen1[i] + beta3 * ReMDen2[i] + beta4 * VPos[i] + beta5 * Pop1[i] + beta6* Pop2[i] + beta7 * Ck1[i] + beta8 * Ck2[i] + beta9 * Deg1[i] + beta10 * Deg2[i]

RR[i] <- exp(log.RR[i])

}

#Other priors

tau.s ~ dgamma(0.5, 0.0005)

alpha ~ dflat()

beta1 ~ dnorm(0,0.00001)

beta2 ~ dnorm(0,0.00001)

beta3 ~ dnorm(0,0.00001)

beta4 ~ dnorm(0,0.00001)

beta5 ~ dnorm(0,0.00001)

beta6 ~ dnorm(0,0.00001)

beta7 ~ dnorm(0,0.00001)

beta8 ~ dnorm(0,0.00001)

beta9 ~ dnorm(0,0.00001)

beta10 ~ dnorm(0,0.00001)

}

#Initial values

list(alpha = 0, beta1 = 0, beta2 = 0, beta3 = 0, beta4 = 0, beta5 = 0, beta6 = 0, beta7 = 0, beta8 = 0, beta9 = 0, beta10 = 0, tau.s=0.5)

References

1 Council., C. P. o. C. C. C. a. t. S. Outline of the integrated regional development of the Yangtze River Delta. *People's Daily* [**http://paper.people.com.cn/rmrb/html/2019-12/02/nw.D110000renmrb_20191202_2-01.htm**](http://paper.people.com.cn/rmrb/html/2019-12/02/nw.D110000renmrb_20191202_2-01.htm) (2019).
